# Global estimates of tuberculosis incidence during pregnancy and postpartum: a rapid review and modelling analysis

**DOI:** 10.1016/S2214-109X(25)00431-0

**Published:** 2026-01-07

**Authors:** Nyashadzaishe Mafirakureva, Anna Cartledge, Isobella Bradshaw, Adrie Bekker, Nicole Salazar-Austin, Sue-Ann Meehan, Landon Myer, Jasantha Odayar, Molebogeng X Rangaka, Peter J Dodd

**Affiliations:** a Sheffield Centre for Health and Related Research, School of Medicine & Population Health, University of Sheffield, Sheffield, UK; b Department of Paediatrics and Child Health, Faculty of Medicine and Health Sciences, Stellenbosch University, Cape Town, South Africa; c Department of paediatrics, Johns Hopkins University School of Medicine, Baltimore, MD, USA; d Desmond Tutu TB Centre, Department of Paediatrics and Child Health, Faculty of Medicine and Health Sciences, Stellenbosch University, Cape Town, South Africa; e Division of Epidemiology & Biostatistics, School of Public Health, University of Cape Town, South Africa; f Institute for Global Health, University College London, London, UK

## Abstract

**Background:**

Despite known maternal, perinatal, and infant health risks of tuberculosis during pregnancy, global estimates of incidence remain scarce. Existing estimates are outdated, and do not include the postpartum period, HIV co-infection, age, or specific changes in risk, limiting our understanding of the true scale of disease in this understudied population.

**Methods:**

In this rapid review and modelling analysis, we estimated the global tuberculosis incidence in pregnant and postpartum women using a population-based modelling approach. We searched MEDLINE and EMBASE, with no date or language limits, and included studies reporting tuberculosis incidence in pregnancy or postpartum with suitable comparison groups; we also used Feb 6, 2025, interim data from the ongoing ORCHID cohort. We combined WHO age and sex-stratified tuberculosis incidence data with country-specific population and fertility data to estimate baseline tuberculosis incidence, and applied systematic review-based risk ratios to account for elevated increased risk during pregnancy and postpartum. Uncertainty in all inputs was propagated using standard error propagation formulae and summarised as mean tuberculosis incidence rates and mean incidence rate ratios (IRRs), each reported with 95% quantile-based uncertainty intervals (UIs).

**Findings:**

We identified 37 studies published between 1996 and 2020, of which three were of sufficient quality to provide data for HIV-negative women. One additional study (ORCHID; Odayar et al, unpublished) provided data for women living with HIV. Compared with non-pregnant women without HIV, tuberculosis IRRs were 1·34 (95% CI 1·17–1·54) during pregnancy and 1·91 (1·53–2·39) during postpartum among HIV-negative women. For women living with HIV, IRRs were 5·73 (95% CI 2·64–10·94) during pregnancy and 3·58 (0·85–9·63) postpartum. We estimated 239 500 pregnant women (95% UI 216 300–262 800) and 97 600 postpartum women (90 100–105 200) developed tuberculosis disease globally in 2023, with HIV contributing to 21·3% (19·8–22·8) and 10·6% (9·9–11·3) of cases, respectively. The WHO African region had the highest incidence (110 600 [95% UI 96 700–124 500] in pregnant women and 40 900 [36 300–45 400] in postpartum women), followed by the South-East Asia region (79 900 [64 100–95 700] in pregnant women and 35 900 [30 800–41 100] in postpartum women).

**Interpretation:**

Pregnant and postpartum women face substantial tuberculosis risk, yet remain under-represented in global estimates. Our findings underscore the need for improved surveillance and targeted interventions to reduce tuberculosis incidence in this group.

**Funding:**

UK Medical Research Council.

## Introduction

Tuberculosis is a major public health threat, responsible for an estimated 1·25 million deaths globally in 2023.^1^ In many high-burden settings, tuberculosis incidence peaks in women (ie, all people who could become pregnant, including those who do not identify as women) during the reproductive years (age 15–49 years, as defined by WHO).^2^, ^3^ Pregnant and postpartum women can have a higher risk of developing tuberculosis disease than non-pregnant women.^4^ Maternal tuberculosis is associated with adverse pregnancy outcomes, including low birthweight, preterm birth, maternal mortality, stillbirth, and infant mortality.^5^ A high incidence of postpartum tuberculosis has been associated with increased maternal and infant mortality, particularly among people living with HIV.^6^ Although tuberculosis preventive treatment (TPT) is not contraindicated in people living with HIV, its use during pregnancy is often scarce due to persistent safety concerns.^7^ Furthermore, diagnosing tuberculosis during pregnancy is challenging; symptoms (eg, shortness of breath) can overlap with pregnancy symptoms, physiological changes in pregnancy can mask tuberculosis symptoms (eg, gestational weight gain), and safety concerns about chest radiographs restrict screening.^7^

The burden of tuberculosis among pregnant and postpartum women is not well characterised. Tuberculosis during pregnancy is rarely reported separately in global surveillance reports (including routine national notifications compiled by WHO), leaving substantial gaps in data and understanding. Pregnancy status is not typically recorded or routinely reported as a tuberculosis programme indicator and tuberculosis data is not recorded in pregnancy registers.^8^ The only modelling study that we identified was published in 2014 and estimated that 216 500 pregnant women developed tuberculosis in 2011, with 41% of cases occurring in Africa; no estimates were included for the postpartum period.^9^ Additionally, these estimates did not account for age-specific variations in tuberculosis incidence, or the elevated risk posed by pregnancy and postpartum periods, particularly in the context of HIV co-infection.

**Research in context**

**Evidence before this study**

The burden of tuberculosis in pregnancy remains poorly quantified despite well recognised risks. We searched PubMed using the terms (“tuberculosis” OR “tubercul*”) AND (“burden*”) AND (“pregnan*” OR “postpartum*”) AND (“global*”) for articles published before May 31, 2025, without language restrictions. The search identified only one study that provided global estimates of active tuberculosis in pregnancy, highlighting a crucial evidence gap. Sugarman and colleagues (2014) used mathematical modelling to project 216 500 cases (95% uncertainty range 192 100–247 000) among pregnant women worldwide in 2011. These estimates do not include the postpartum period, nor do they account for increased risks during pregnancy or due to HIV. Our rapid review, supported by a recent systematic review on tuberculosis infection in pregnancy (Morton and colleagues, 2024), identified several studies assessing the risk of tuberculosis during pregnancy and postpartum in different settings. Earlier retrospective studies from the Dominican Republic (Espinal and colleagues, 1996) and Malawi (Crampin and colleagues, 2004) found no increased risk in these periods. By contrast, more recent and larger registry-based cohort studies, including Zenner and colleagues (2012) in the UK and Jonsson and colleagues (2020) in Sweden, reported increased risks, with incidence rate ratios ranging from 1·30 to 1·40 during pregnancy and 1·90 to 2·00 during postpartum compared with women who are not pregnant. Similarly, Rendell and colleagues (2016) found, in Mongolia, a 1·3-fold increased risk among pregnant women compared with the general population. Notably, a study by Odayar and colleagues (2018) in South Africa found no increased tuberculosis risk among women living with HIV during pregnancy or postpartum compared with preconception.

**Added value of this study**

Our work advances this scarce evidence base by generating updated incidence estimates that include both pregnancy and the postpartum period globally. We improve on previous estimates by incorporating recent age and sex-stratified tuberculosis incidence data and adjusting for increased tuberculosis risk associated with pregnancy, the postpartum period, and HIV, on the basis of a systematic review of existing literature and modelling.

**Implications of all the available evidence**

Available evidence collectively shows that pregnant and postpartum women have a substantial tuberculosis burden, with potentially serious consequences for maternal, perinatal, and infant health outcomes. These findings underscore the urgent need to strengthen tuberculosis surveillance within maternal health programmes, implement targeted screening and treatment interventions in high-burden settings during the pregnant and postpartum periods, and integrate pregnancy-specific indicators into global tuberculosis monitoring frameworks to better address this neglected aspect of the epidemic.

Since the 2014 study's global estimates,^9^ WHO age and sex-specific estimates of tuberculosis incidence have become available,^10^ and additional studies have examined the risk of tuberculosis during pregnancy.^3^ In this modelling study we aimed to update and expand tuberculosis incidence estimates during pregnancy and the postpartum period. To do this, we first used a rapid review and meta-analysis methodology to quantify the incidence rate ratios (IRRs) for tuberculosis during pregnancy and the postpartum period. Furthermore, we sought from our contacts additional cohort data to quantify the risk of tuberculosis among pregnant and postpartum women living with HIV. Finally, we used age-specific tuberculosis incidence data and applied these IRRs within an established mathematical modelling framework to estimate global and country-specific tuberculosis incidence in pregnant and postpartum women by age and HIV infection status.

## Methods

### Study design

We did a rapid review to identify and synthesise evidence on the risk of tuberculosis during pregnancy and postpartum. The research question was developed using the population, exposure, comparator, outcome, and study (PECOS) framework. The rapid review question was as follows: among women in the reproductive age group (population), what is the relative risk of tuberculosis disease (outcome) during pregnancy and the postpartum period (exposure) compared with non-pregnant or non-postpartum women (comparator), observed and reported in studies (or study)? The rapid review protocol was registered with PROSPERO (identification number CRD42018111690).

### Search strategy

Two independent reviewers (AC and IB) did systematic searches on Sept 28, 2018, in MEDLINE and EMBASE databases via OVID, without applying any date or language restrictions. The searches aimed to capture studies related to “pregnancy or postpartum” as exposures, “tuberculosis” as the outcome, and “observational study” as the study design. The search terms for “pregnancy or postpartum” and “tuberculosis” were adapted from similar Cochrane reviews. For the term “observational study”, we applied filters recommended by the Scottish Intercollegiate Guidelines Network (SIGN)^11^ for observational studies.

To ensure comprehensive coverage, hand-searching of the references and citations of articles included after full-text review was done. An update of the MEDLINE and EMBASE searches was conducted by a single reviewer (NM) on Feb 19, 2024, to include any newer evidence. Professional networks were consulted to identify any data or studies missed by the search process.

### Study characteristics and eligibility criteria

For the rapid review, we aimed to include three types of studies. Type 1 studies were longitudinal studies reporting tuberculosis incidence during pregnancy or postpartum, either with a non-pregnant control group or controlled internally through case series designs. Type 2 studies were cross-sectional studies reporting tuberculosis prevalence during pregnancy or postpartum, compared with a non-pregnant control group, and reporting odds ratios (ORs). Type 3 studies were cross-sectional studies reporting the prevalence of pregnancy or postpartum status in tuberculosis cohorts, which reported ORs or provided data required to calculate ORs. Type 3 studies were also included if post-hoc control group construction seemed feasible, based on publicly available population fertility data. Studies without an appropriate control group, or where one could not be reasonably constructed, were excluded, as were studies focusing on latent tuberculosis as the main domain of interest was active tuberculosis.

### Data extraction and analysis

Data extracted from selected studies included study location and period, study design, population characteristics, control group details, participant demographics (ie, HIV status and age), tuberculosis screening and diagnostic approaches, tuberculosis case definition, proportion of cases with bacteriological confirmation, and postpartum period definition. Discrepancies between reviewers (AC and IB) were addressed through discussion with a third independent reviewer (PJD) until consensus was reached. All data were cross-checked for accuracy and consistency. The US National Institutes of Health (NIH) Quality Assessment Tool for Observational Cohort and Cross-Sectional Studies^12^ was used to assess the risk of bias in individual studies. We report rapid review findings following PRISMA^13^ guidelines. We provide a narrative summary and quality appraisal for the included studies. Studies rated as good (ie, low risk of bias) were included in the quantitative synthesis.

### Meta-analysis

The primary outcome of the rapid review was the IRR for tuberculosis during pregnancy and the postpartum period compared with non-pregnant or non-postpartum women. ORs from cross-sectional studies were converted into IRRs to facilitate comparison, with a preference for adjusted ORs when available. Effect sizes from individual studies were pooled using inverse variance weighting to generate summary IRRs. Heterogeneity between studies was assessed with *I*^2^ and τ^2^ statistics. Forest plots were generated to visualise the results.

### IRRs for people living with HIV

A single analysis from the ongoing obesogenic origins of maternal and child metabolic health involving dolutegravir (ORCHID) study (NCT04991402; Odayar et al, unpublished)^14^ was provided by LM and JO to quantify the risk of tuberculosis among pregnant and postpartum women living with HIV. We used an interim Feb 6, 2025, dataset from this prospective cohort, comprising 1920 pregnant women (804 [42%] living with HIV), enrolled in Cape Town, South Africa; these unpublished data included individual-level information on tuberculosis incidence during pregnancy and postpartum. We constructed aggregated person-time at risk and incident tuberculosis event counts for women in the not-pregnant, pregnant, and postpartum periods, and used these to compute rate ratios relative to not-pregnant periods.

### Modelling tuberculosis incidence during pregnancy and postpartum

**Figure 1:**
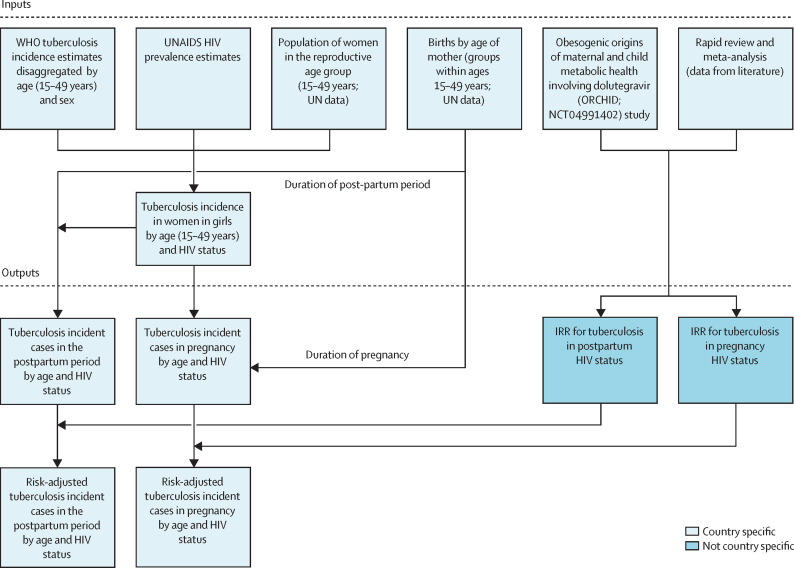
Modelling logic overview showing data sources and how they were combined into outputs IRR=incidence rate ratio.

The incidence of tuberculosis was estimated using a combination of 2023 WHO data on age and sex-disaggregated tuberculosis incidence,^15^ and UN World Population Prospects 2024^16^ estimates of population and fertility (figure 1). The number of pregnancies was approximated using livebirth estimates by maternal age (groups within ages 15–49 years) from the UN World Population Prospects 2024. To calculate person-time at risk, we estimated exposure separately for pregnancy and postpartum, using an average duration of 9 months for pregnancy and 3 months for postpartum—consistent with evidence that suggests excess risk of tuberculosis soon after giving birth.^17^

The baseline tuberculosis incidence rate in women of reproductive age was calculated using WHO tuberculosis incidence data and UN population estimates for females aged 15–49 years; this provided the annual tuberculosis incidence rate, which includes both women living with and without HIV. To account for the differential risk of tuberculosis by HIV status, the incidence rate was disaggregated for women living with HIV and those not living with HIV, using UNAIDS estimates of HIV prevalence among pregnant women and the relative risk of tuberculosis among women living with HIV.^18^ The relative risk of tuberculosis among women living with HIV (ie, IRRs) was estimated by comparing the odds of tuberculosis in HIV-positive women of reproductive age (derived from WHO tuberculosis burden data)^16^ and the odds of HIV in women of reproductive age (derived from UNAIDS HIV prevalence data),^18^ with adjustments for uncertainty and missing data. The disaggregated tuberculosis incidence estimates were then multiplied by the person-time at risk to estimate the total annual number of pregnant and postpartum women with active tuberculosis by HIV status.

Finally, to reflect the increased risk of tuberculosis during pregnancy and the postpartum period, we applied IRRs derived from our rapid review and meta-analysis. These IRRs, stratified by HIV status, adjusted the tuberculosis incidence estimates to capture the heightened vulnerability of women during pregnancy and postpartum.

Our analysis propagated reported uncertainty from tuberculosis incidence estimates, HIV data, population and birth data, and IRRs using standard error propagation formulae (details in the appendix p 2) using R software (version 4.5.0).

### Role of the funding source

The funder of the study had no role in study design, data collection, data analysis, data interpretation, writing of the manuscript, or decision to submit for publication.

## Results

The database search identified 2035 records, of which 1997 were excluded after deduplication and screening of titles and abstracts. Of 37 articles selected for a full-text review, 31 were excluded, leaving six articles for qualitative assessment (appendix p 4). Most records (65%; 20 of 31) were excluded for not having a relevant control group. The six studies included in the qualitative assessment were published between 1996 and 2020, spanning an observation period from 1992 to 2014, and reported data from six countries: the Dominican Republic,^19^ Malawi,^20^ Mongolia,^21^ South Africa,^22^ Sweden,^4^ and the UK (table 1).^17^ The study designs comprised two case-control studies and four retrospective cohort studies. Two studies included data on HIV,^20^, ^22^ with one focusing exclusively on HIV.^22^ Five studies reported data on both pregnancy and postpartum (defined as within 6 months after delivery),^4^, ^17^, ^19^, ^20^, ^22^ and one focused exclusively on pregnancy.^21^ The quality of the six studies for our question was rated as poor (one study),^20^ fair (one study),^19^ and good (four studies)^4^, ^17^, ^21^, ^22^ on the basis of the number of positive responses to the NIH's Quality Assessment Tool questions.^12^ In addition to the six studies identified through literature review, a dataset from a cohort of women living with HIV from an ongoing trial (ORCHID [Odayar et al, unpublshed data; NCT04991402])^14^ in South Africa was found through professional networks.

**Table 1:** Summary of included studies

	**Study years**	**Country**	**Population and setting**	**Study type**	**Control group**	**HIV status**	**Mean age**	**Tuberculosis case definition**	**Population; person-years of data**	**Quality assessment**
**Studies in populatioons without HIV**										
Crampin et al, 2004^20^	1996–2001	Malawi	Male and female patients with tuberculosis identified via enhanced passive surveillance	Case control	Field-based random sampling of the community	Cases: 68% positive; controls: 14% positive	Not available	Positive smear with culture or biopsy	..	Poor
Espinal et al, 1996^19^	1992–94	Dominican Republic	Women aged 15–44 years	Case control	Recruited at the National Laboratory of Public Health when they presented for anonymous HIV testing	Cases: 6% positive; controls: 50% positive	29 years	Must have all typical signs and symptoms of tuberculosis, >1 smear of respiratory tract secretions positive for acid-fast bacilli, and no history of previous tuberculosis	..	Fair
Odayar et al,!y* 2018^22^	2013–14	South Africa	HIV-positive women during pregnancy and postpartum, aged ≥18 years at a large public sector primary health care facility that provides antenatal care to >4000 women annually	Retrospective cohort study	Women up to 18 months preconception	All positive	29 years at enrolment (IQR 26–33)	Positive sputum smear microscopy, Xpert MTB/RIF assay (Cepehid, Sunnyvale, CA, USA), tuberculosis culture, and chest x-ray abnormalities consistent with tuberculosis	1513; 4116	Good
Rendell et al, 2016^21^	2013	Mongolia	Pregnant women diagnosed with tuberculosis from central tuberculosis dispensaries	Retrospective cohort study	General population	Population prevalence <0·1%	27 years (SD 5·6, range 18–43)	Based on microbiological results or symptoms and chest x-ray findings	2 113 969; 2 099 459	Good
Zenner et al, 2012^17^	1996–2008	UK	All women with pregnancies that occur within the time period studies	Retrospective cohort study	Self-controlled case series	Not available	29·5 years (range 13–50)	Positive sputum culture, presence of clinical or radiological signs, and symptoms	192 801; 1 745 834	Good
Jonsson et al, 2020^4^	2005–13	Sweden	Women aged 15–49 years who had given birth in Sweden	Retrospective cohort study	Women who were neither pregnant nor postpartum	Not available	15–49 years	Laboratory confirmation (culture or microscopy and PCR) and clinical or radiological findings	649 342; 552 8122	Good
**Studies in populations with HIV**										
ORCHID (Odayar et al, data unpublished: NCT04991402), 2025^14^	2021–ongoing; dataset taken Feb 6, 2025	South Africa	Pregnant women and pregnant women living with HIV, followed up from ≤18 weeks gestational age to 24 months postpartum	Prospective observtional study	Women who were neither pregnant nor postpartum	42% positive	28 years (IQR 23–32)	Microbiological confirmation or clinical and/or radiological findings	1920; 19 534 314	Not quality rated

Six published studies in populations without HIV were included in the qualitative review. Only three of these studies in populations without HIV (Jonsson et al,^4^ Rendell et al,^21^ and Zenner et al^17^) were included in the primary meta-analysis.

* Excluded from the meta-analysis because of potential confounding due to antiretroviral therapy and tuberculosis preventive treatment.

**Figure 2:**
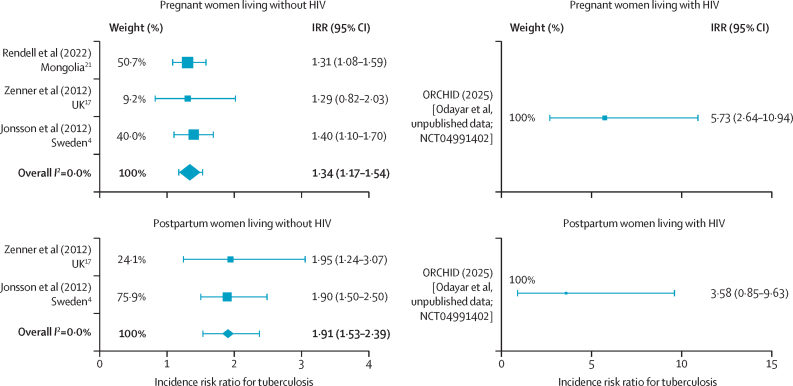
IRRs for tuberculosis during pregnancy and postpartum, with and without HIV Weight represents the percentage of the total inverse variance contributed by each study to the meta-analysis. Squares show individual study effect sizes (size proportional to each study's relative weight in the meta-analysis). Diamonds represent pooled estimates. Error bars show 95% uncertainty intervals. IRRs=incidence rate ratios.

Three studies were included in the primary meta-analysis: Jonsson and colleagues,^4^ Rendell and colleagues,^21^ and Zenner and colleagues.^17^ We obtained IRR estimates for pregnant and postpartum women living with HIV from a dataset from an ongoing study (ORCHID [Odayar et al, unpublished data; NCT04991402]; 2025) for comparison.^14^ Odayar and colleagues' (2018)^22^ study was excluded for potential confounding due to antiretroviral therapy and tuberculosis preventive treatment. Crampin and colleagues^20^ and Espinal and colleagues^19^ were excluded as they were judged to be of poor methodological quality for our purpose in the risk of bias assessment, including inconsistent population selection, unclear eligibility criteria, lack of sample size justification, unclear temporal relationship between exposure and outcome, poorly defined outcome measures, and absence of assessor blinding (appendix p 5). The summary IRR for tuberculosis among HIV-negative women was 1·34 (95% CI 1·17–1·54) during pregnancy and 1·91 (1·53–2·39) during postpartum compared with non-pregnant and non-postpartum periods (figure 2). The IRR for tuberculosis among women living with HIV quantified from ORCHID (Odayar et al, unpublshed data; NCT04991402)^14^ at the time of this analysis was 5·73 (95% CI 2·64–10·94) during pregnancy and 3·58 (0·85–9·63) during postpartum (figure 2).

**Figure 3:**
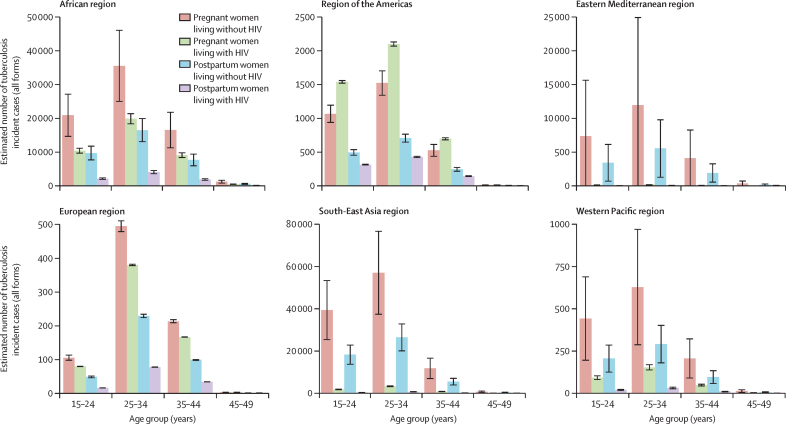
Age and HIV status disaggregated tuberculosis incidence estimates during pregnancy and postpartum Error bars represent 95% uncertainty intervals around the incidence estimates. All forms=total tuberculosis incidence (pulmonary and extrapulmonary disease).

The global estimated person-time at risk during 2023 was 101 158 100 person-years (95% uncertainy interval [UI] 97 160 600–105 155 700) during pregnancy and 32 966 700 person-years (31 663 900–34 269 500) during the postpartum period. An estimated 239 500 (95% UI 216 300–262 800) pregnant women and 97 600 (90 100–105 200) postpartum women developed active tuberculosis in 2023. In total, this represents 9% of tuberculosis incidence among women aged 15 years or older, globally. The proportions of tuberculosis cases coinfected with HIV were 21·3% (95% UI 19·8–22·8) during pregnancy and 10·6% (0·9–11·3) during postpartum (table 2). The highest burden was observed in the WHO African region, with 110 600 (95% UI 96 700–124 500) pregnant women and 40 900 (36 300–45 400) postpartum women affected (table 2). The WHO South-East Asia region also carried a substantial burden, with 79 900 (64 100–95 700) pregnant women and 35 900 (95% UI 30 800–41 100) postpartum women developing active tuberculosis. Most tuberculosis cases were observed in the 25–34 years age groups (when most people have children) across all WHO regions (figure 3).

**Table 2:** Global and regional estimates of tuberculosis incidence during pregnancy and postpartum disaggregated by HIV status

	**Pregnancy**			**Postpartum**		
	All pregnant women	Women living without HIV*	Women living with HIV†	All women in the postpartum period	Women living without HIV*	Women living with HIV†
African region	110 600 (96 700–124 500)	70 600 (57 300–83 900)	40 000 (38 200–41 800)	40 900 (36 300–45 400)	32 700 (28 400–36 300)	8100 (7600–8700)
Region of the Americas	7500 (6800–8100)	3100 (2900–3400)	4300 (4300–4400)	2300 (2100–2500)	1400 (1400–1500)	900 (900–900)
Eastern Mediterranean region	24 100 (15 600–32 700)	23 700 (7600–39 800)	400 (400–500)	11 100 (8300–13 900)	11 000 (5800–16 200)	100 (100–100)
European region	2900 (2500–3300)	2200 (1900–2400)	800 (800–800)	1200 (1000–1300)	1000 (900–1100)	200 (200–200)
South-East Asia region	79 900 (64 100–95 700)	75 500 (57 500–93 400)	4400 (4200–4600)	35 900 (30 800–41 100)	35 000 (29 200–40 900)	900 (800–1000)
Western Pacific region	14 600 (9800–19 300)	12 800 (8000–17 500)	1800 (1700–1900)	6300 (4700–7800)	5900 (4400–7500)	400 (300–400)
Total	239 500 (216 300–262 800)	187 800 (159 900–215 800)	51 700 (49 900–53 500)	97 600 (90 100–105 200)	87 100 (78 000–96 200)	10 500 (9900–11 100)

Data are mean (95% uncertainty interval).

* Data from three published studies: Jonsson et al,^4^ Rendell et al,^21^ Zenner et al.^17^

† Data from the ORCHID study (Odayar et al, unpublished data; NCT04991402).^14^

## Discussion

Our study responds to a key evidence gap by providing to our knowledge the first updated global estimates of tuberculosis incidence during pregnancy and the postpartum period since the only previous analysis published a decade ago.^9^ Our findings highlight that tuberculosis is a substantial public health burden and further delineate the disproportionate burden attributable to HIV co-infection in this understudied population. Our review and meta-analysis provide evidence for increased risk of tuberculosis incidence during pregnancy and postpartum. Combining these IRRs with current estimates of age-specific fertility and tuberculosis incidence suggests a global tuberculosis incidence of 239 500 cases during pregnancy and 97 600 during postpartum (taken as 3 months). HIV co-infection accounted for 21·3% of tuberculosis cases during pregnancy and 10·6% during postpartum, emphasising the continued role of HIV as a crucial risk modifier. These overall findings add weight to the growing global consensus around the need to include pregnant and postpartum women in research, including tuberculosis prevention trials, and highlight the need to prioritise routine tuberculosis screening and testing among these women.

To put our global estimates into context, Cresswell and colleagues^23^ estimated a global mortality of 253 000 from maternal sepsis for the period 2009–20, highlighting it as a major contributor to maternal morbidity and mortality worldwide. The annual tuberculosis incidence estimates we derived in this study among pregnant and postpartum women are of a comparable magnitude. Beyond maternal outcomes, the implications of tuberculosis for child health are profound. More than 1 million children are estimated to develop tuberculosis globally every year,^1^ and close contact between mothers and neonates is probably a substantial driver of tuberculosis transmission in these youngest children, who face the highest risk of progression to severe and disseminated disease. The overlap of maternal and paediatric tuberculosis highlights the dual burden borne by families and the potential for maternal tuberculosis to act as a key reservoir of infection within households.

Our meta-analysis indicates that tuberculosis risk is elevated during women's reproductive stages, even among HIV-negative women, with IRRs of 1·34 during pregnancy and 1·91 during postpartum. The higher postpartum IRR could in part reflect underdiagnosis during pregnancy, a period in which tuberculosis is notoriously difficult to detect. Symptoms such as fatigue, mild cough, and weight changes often overlap with normal gestation or other infectious diseases,^24^ and imaging—an essential diagnostic tool—is frequently restricted due to fetal safety concerns.^7^ These diagnostic challenges likely delay detection, with many cases that develop during pregnancy only being recognised during postpartum, contributing to the apparent surge in risk after delivery. These issue highlight not only the biological vulnerability of women during these stages but also the crucial need for improved, pregnancy-adapted diagnostic strategies to ensure timely identification and treatment. Among women living with HIV, the higher IRRs of 5·73 during pregnancy and 3·58 during postpartum reflect the compounded susceptibility due to immunosuppression and underscore the urgent need for integrated HIV and tuberculosis care during these periods.

Our estimates underscore the intersection of tuberculosis and HIV in shaping maternal and infant health risks. In the WHO African region, we estimate that overall 32% of tuberculosis incidence among pregnant and postpartum women occurs in women living with HIV. This finding has two important implications. First, HIV co-infection increases the risk of maternal morbidity and mortality from tuberculosis, exacerbating the already high susceptibility during pregnancy and postpartum. Second, children born to women living with HIV are not only more likely to be exposed to maternal tuberculosis but might themselves be HIV-exposed or HIV-infected, placing them at particularly high risk of poor outcomes. This dual vulnerability—maternal HIV-tuberculosis co-infection and paediatric HIV exposure—represents a crucial driver of infant morbidity and mortality. These findings highlight the need for integrated maternal, newborn, and child health programmes that incorporate tuberculosis and HIV prevention, early detection, and treatment as essential components of care.

Tuberculosis during pregnancy carries increased risks of premature birth, low birthweight, stillbirth and infant mortality, and vertical HIV transmission.^25^ WHO guidelines recommend integrated, decentralised, and family-centred approaches to tuberculosis care during pregnancy and do not consider pregnancy a contraindication for TPT; the 6-month isoniazid regimen is recommended for all people living with HIV, including pregnant women.^26^, ^27^ Although our study did not model the effect of TPT, the high burden of tuberculosis we report underscores the urgent need for further studies that quantify the potential benefits of TPT in this high-risk population. Existing modelling from South Africa suggests that immediate TPT for people living with HIV during pregnancy could reduce maternal and infant deaths.^28^ Additional work is needed to define the optimal timing for implementation and to characterise the safety of alternative regimens, particularly in the context of drug-resistant tuberculosis. Moreover, diagnosis of tuberculosis during pregnancy remains challenging: the WHO four-symptom screen shows suboptimal sensitivity in pregnancy.^29^ Improved screening tools, safety data on shorter regimens, and guidance on managing drug-resistant tuberculosis are urgently required. Furthermore, reproductive health assessment and contraception should be prioritised during anti-tuberculosis treatment to avoid unintended pregnancies that can complicate management and outcomes.

Although previous studies have examined tuberculosis risk in pregnancy, our analysis provides some important advances. First, our work goes beyond that of a recent systematic review^3^ that independently identified similar evidence, but did not include a formal synthesis or HIV-specific risks, to produce quantitative estimates of risk during pregnancy and the postpartum. Second, our modelling advances upon the work by Sugarman and colleagues^9^ by encompassing the postpartum period as a distinct risk phase, accounting for pregnancy and postpartum-associated changes in tuberculosis risk, integrating HIV co-infection as an additional risk factor, and using age-disaggregated tuberculosis incidence data as a starting point. These methodological refinements probably explain why our estimate of 239 500 pregnancy-associated cases in 2023 exceeds Sugarman's 2011 projection (216 500 cases),^9^ despite an 8% decline in global tuberculosis incidence during this interval.^1^ This discrepancy suggests systematic underestimation in previous models that omitted co-infection with HIV and pregnancy-related risk elevation. Our approach enables stratification by maternal age and HIV status, which is important for planning prevention efforts in settings with high HIV and tuberculosis burdens.

Despite the important insights from our findings, our analysis is constrained by existing evidence gaps. Only six published studies were identified for the rapid review: most are from more than 8 years ago and from low tuberculosis burden settings. Although the two largest studies based on European electronic health records showed concordant results,^4^, ^17^ their generalisability to high-burden settings might be limited (due to differences in tuberculosis epidemiology, HIV prevalence, and health system capacity), resulting in higher potential for bias in our estimates at country-level. For women living with HIV, we derived IRRs using data from the ORCHID cohort (NCT04991402; Odayar et al, unpublished data)^14^ in South Africa; however, these findings should be interpreted with caution, as the dataset is unpublished and based on interim analyses restricted to the Western Cape of South Africa, which differs from other provinces in terms of HIV prevalence and fertility patterns. Although these limitations warrant caution, IRRs represented just one of many model inputs, most of which were country-specific. Further studies should be undertaken to strengthen and nuance our understanding of relative tuberculosis risks during pregnancy and postpartum. Replicating the decade-old UK study^17^ or leveraging consolidated provincial health data from South Africa's Western Cape Provincial Health Data Centre^30^ offer clear opportunities to expand this evidence base.

The use of livebirths to estimate person-time at risk neglects scenarios such as multiple births (~3% globally),^31^ stillbirths (~1·4% of pregnancies),^32^ or abortions. Although stillbirths and multiple births would probably have minimal effect on overall estimates, the effect of abortions remains unquantified due to scarce data on abortion timing and frequency, and a complete absence of evidence regarding whether tuberculosis incidence rate ratios apply equivalently to terminated pregnancies.

More importantly, we assumed that the same IRRs applied in all regions and regardless of age. The IRRs are largely based on cohorts from low tuberculosis-incidence European countries and could differ in other settings. These IRRs likely capture biological and social associations, and social associations between pregnancy and tuberculosis risk are expected to vary regionally. Additional data from diverse high tuberculosis-incidence settings would help to address this limitation. Source studies used postpartum periods ranging from 3 to 6 months, so we adopted a conservative 3-month duration for our definition of postpartum person-time at risk; in effect, we assumed that increases in tuberculosis risk seen in longer studies is concentrated in the first 3 months. Longer definitions of postpartum with these IRRs, or use of IRRs specific to the first 3 months postpartum, would each generate larger estimates of postpartum tuberculosis incidence.

Currently, modelling is required to estimate the incidence of tuberculosis in pregnant and postpartum women because direct data from surveillance systems are lacking. Although WHO guidelines recommend screening all pregnant women living in high-burden countries and/or those living with HIV,^26^ tuberculosis screening is not systematically recorded in many countries. Routinely capturing pregnancy status in tuberculosis registers and integrating screening into antenatal services would generate empirical data to validate and refine estimates while identifying people in need of care—particularly in low-income countries, in which high fertility rates^33^ and tuberculosis prevalence intersect.^8^ Practical strategies include introducing pregnancy status fields in national tuberculosis registers, linking tuberculosis and maternal health information systems, and training health workers to record this information consistently. Registers that track pregnancy outcomes for TPT and anti-tuberculosis treatment would generate essential safety data to inform policy. Although modelled estimates remain important for planning and advocacy, the strengthening of routine data collection and systematic inclusion of pregnant women in research on tuberculosis screening, diagnosis, treatments, and vaccines^33^, ^34^ are crucial to close the current evidence gap and help ensure equitable care delivery.

Pregnant and postpartum women are likely to be at increased risk of tuberculosis and large numbers develop tuberculosis globally, affecting maternal health and neonatal and infant outcomes. More effort is needed to strengthen epidemiological and pharmacological surveillance, and to integrate tuberculosis screening in antenatal care.

### Contributors

### Data sharing

All code and data to reproduce this analysis are publicly available on GitHub: https://github.com/petedodd/pregtb.

## Declaration of interests

PJD was supported by a fellowship from the UK Medical Research Council (MR/P022081/1). All other authors declare no competing interests.
