# Supplementary material for: Global estimates of tuberculosis incidence during pregnancy and postpartum: a rapid review and modelling analysis
Source: Lancet Glob Health. 2026 Jan 7;14(3):e337–46. doi: 10.1016/S2214-109X(25)00431-0 (PMC12916327; doi:10.1016/S2214-109X(25)00431-0)
Supplement: Supplementary appendix [file mmc1.pdf]

THE LANCET
Global Health

Supplementary appendix

This appendix formed part of the original submission and has been peer reviewed. We post it as supplied by the authors.

Supplement to: Mafirakureva N, Cartledge A, Bradshaw I, et al. Global estimates of tuberculosis incidence during pregnancy and postpartum: a rapid review and modelling analysis. *Lancet Glob Health* 2026; published online Jan 7. https://doi.org/10.1016/S2214-109X(25)00431-0.

Global estimates of tuberculosis incidence during pregnancy and postpartum: a rapid review and modelling analysis

Authors

Nyashadzaishe Mafirakureva[1], Anna Cartledge[1], Isobella Bradshaw[1], Adrie Bekker[2], Nicole Salazar-Austin[3], Sue-Ann Meehan[4], Landon Myer[5], Jasantha Odayar[5], Molebogeng X. Rangaka[6], Peter J. Dodd[1]

Affiliations:

1. Sheffield Centre for Health and Related Research (SCHARR), School of Medicine & Population Health, University of Sheffield, Sheffield, UK
2. Department of Paediatrics and Child Health, Faculty of Medicine and Health Sciences, Stellenbosch University, Cape Town, South Africa.
3. Department of Pediatrics, Johns Hopkins University School of Medicine, Baltimore, MD, USA
4. Desmond Tutu TB Centre, Department of Paediatrics and Child Health, Faculty of Medicine and Health Sciences, Stellenbosch University, Cape Town, South Africa
5. Division of Epidemiology & Biostatistics, School of Public Health, University of Cape Town, South Africa.
6. Institute for Global Health, University College London, London, UK

Supplementary Methods **2**
IRRs for tuberculosis during pregnancy and the postpartum period among women living with HIV 2
IRRs for TB given HIV 2
Uncertainty propagation 2
Supplementary Results **4**
Studies selected for the rapid review 4
Quality assessment for studies selected for the rapid review 5
Sensitivity analysis meta-analysis 6
Population and births 7
Person-time 7
Estimates in high TB burden settings 7
Global distribution of tuberculosis burden during pregnancy and postpartum 10
Country estimates 12
References **21**

Supplementary Methods

IRRs for tuberculosis during pregnancy and the postpartum period among women living with HIV

Data from the Obesogenic oRigins of maternal and Child metabolic Health Involving Dolutegravir (ORCHID) study (NCT 04991402),[1,2] was used to estimate the IRR for TB among women living with HIV. Details of the study have been published elsewhere.[1] In short, the ORCHID study is a prospective observational study taking place in Cape Town, South Africa. The study is following up 1,920 women (1,116 living with HIV, 804 HIV seronegative) from ≤18 weeks gestational age to 24 months postpartum and collected information on TB diagnoses prior to conception of index pregnancy and during pregnancy or within 6 months postpartum.

To calculate incidence and IRRs we first calculated person times during pregnancy, postpartum and not pregnant or postpartum. Person-time during pregnancy was defined as time since conception for those with a valid delivery date and time to maximum available date for those still pregnant. Person-time postpartum was defined as time since delivery for those with a valid delivery. Person-time for women who were neither pregnant nor postpartum was calculated as time from birth to most recent conception. Restricting the analysis to women living with HIV (42%; 804/1,920), the total person-time was 8,364,234 for periods when they were neither pregnant nor postpartum, 211,861 during pregnancy, and 157,764 during the postpartum period. The observed number of TB cases were 63 during periods when neither pregnant nor postpartum, 9 during pregnancy, and 3 during the postpartum period.The estimated IRR for TB among women living with HIV was 5.73 (95% CI: 2.64-10.94) during pregnancy and 3.58 (95% CI: 0.85-9.63) during the postpartum period.

IRRs for TB given HIV

The increased risk of tuberculosis (TB) among people living with HIV (PLHIV) was quantified using the Incidence Rate Ratio (IRR), which compares the odds of TB in HIV-positive individuals to the odds of HIV in the general population. Specifically, IRR was calculated as:

$$IRR = \frac{HIV\ prevalence\ in\ women\ of\ reproductive\ age\ with\ TB\ /\ (1 - HIV\ prevalence\ in\ women\ of\ reproductive\ age\ with\ TB)}{HIV\ prevalence\ in\ women\ of\ reproductive\ age\ /\ (1 - HIV\ prevalence\ in\ women\ of\ reproductive\ age\ )}$$

Data on HIV prevalence in women of reproductive age with TB was based on WHO data on age- and sex-disaggregated TB incidence,[3] while data on HIV prevalence in women of reproductive age was based on UNAIDS data.[4]

Uncertainty propagation

We systematically propagated uncertainty from all input parameters using reported confidence intervals or low/high bounds using standard methods.
For multiplicative relationships,

$$A = B \times C$$

we computed the standard deviation representing uncertainty in the new quantity as

$$\sigma_A = A \times \sqrt{\left(\frac{\sigma_B}{B}\right)^2 + \left(\frac{\sigma_C}{C}\right)^2}.$$

For additive relationships,

$$A = B + C$$

we computed the standard deviation representing uncertainty in the new quantity as

$$\sigma_A = \sqrt{\sigma_B{}^2 + \sigma_C{}^2}.$$

The resulting standard deviation was then used to construct 95% uncertainty intervals (UI) as:

$$UI_A = [A - 1.96 * \sigma_A \,;\, A + 1.96 * \sigma_A].$$

Supplementary Results

Studies selected for the rapid review

Figure S1. PRISMA flow diagram showing the selection of studies included in the rapid review. ART, antiretroviral therapy.

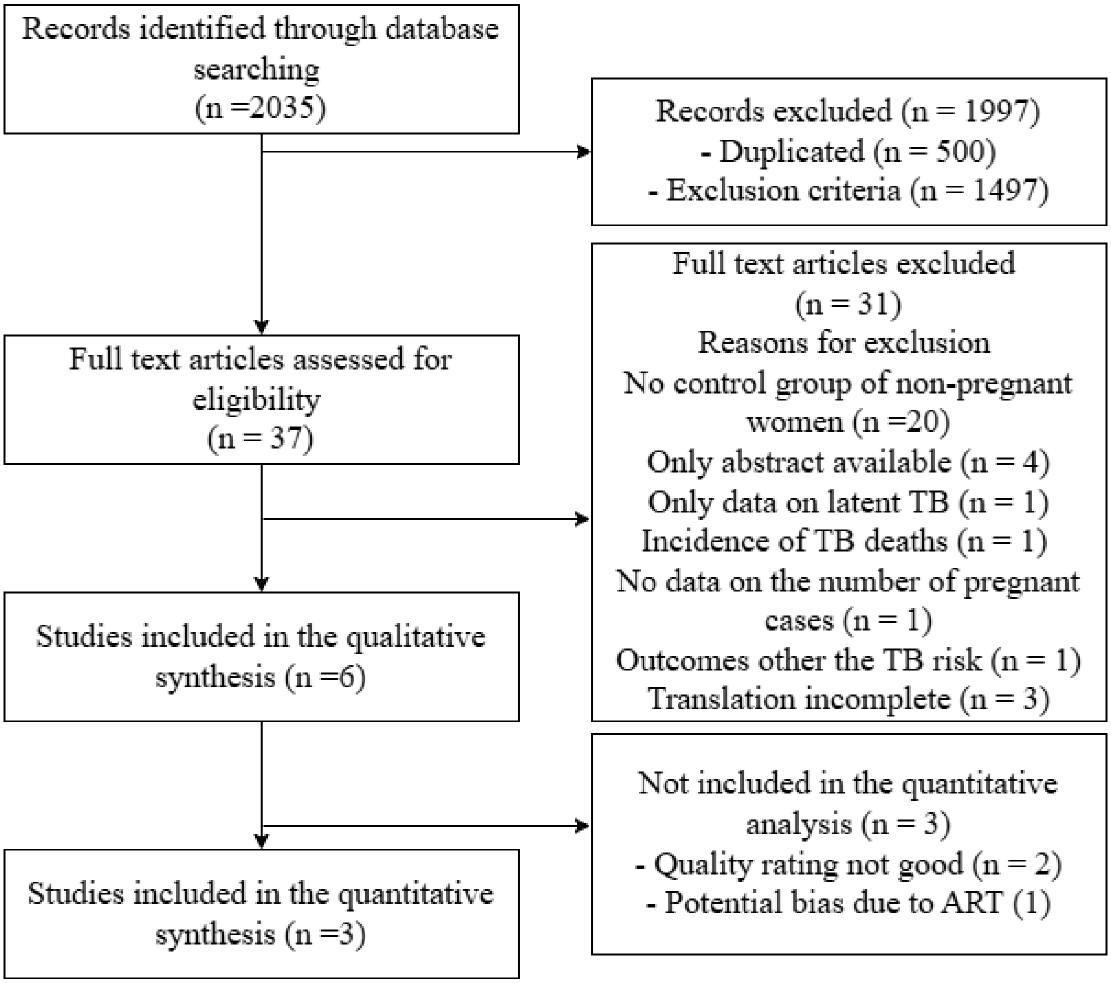

Quality assessment for studies selected for the rapid review

Tables S1. Risk of bias assessment for studies included in the qualitative synthesis using the NIH's Quality Assessment Tool for Observational Cohort and Cross-Sectional Studies.[5] N, No; NA, not applicable; NR, not reported; Y, Yes.

#	NIH's Quality Assessment Tool questions	Crampin, 2004[6]	Espinal, 1996[7]	Odayar, 2018[8]	Rendell, 2016[9]	Zenner, 2012[10]	Jonsson, 2020[11]
1	Was the research question or objective in this paper clearly stated?	Y	Y	Y	Y	Y	Y
2	Was the study population clearly specified and defined?	N	Y	Y	Y	Y	Y
3	Was the participation rate of eligible persons at least 50%?	Y	Y	Y	Y	Y	Y
4	Were all the subjects selected or recruited from the same or similar populations (including the same time period)? Were inclusion and exclusion criteria for being in the study prespecified and applied uniformly to all participants?	N	N	Y	Y	Y	Y
5	Was a sample size justification, power description, or variance and effect estimates provided?	N	N	N	N	Y	Y
6	For the analyses in this paper, were the exposure(s) of interest measured prior to the outcome(s) being measured?	N	N	Y	N	Y	Y
7	Was the timeframe sufficient so that one could reasonably expect to see an association between exposure and outcome if it existed?	Y	Y	N	Y	Y	Y
8	For exposures that can vary in amount or level, did the study examine different levels of the exposure as related to the outcome (e.g., categories of exposure, or exposure measured as continuous variable)?	NA	NA	NA	NA	NA	NA
9	Were the exposure measures (independent variables) clearly defined, valid, reliable, and implemented consistently across all study participants?	Y	Y	Y	N	Y	Y
10	Was the exposure(s) assessed more than once over time?	NA	NA	NA	NA	NA	Y
11	Were the outcome measures (dependent variables) clearly defined, valid, reliable, and implemented consistently across all study participants?	N	Y	Y	Y	Y	Y
12	Were the outcome assessors blinded to the exposure status of participants?	N	N	N	N	N	N
13	Was loss to follow-up after baseline 20% or less?	NR	NR	NR	Y	NR	NR
14	Were key potential confounding variables measured and adjusted statistically for their impact on the relationship between exposure(s) and outcome(s)?	Y	N	Y	N	Y	Y
	**YES**	5	6	8	7	10	11

	**Quality Rating**	Poor	Fair	Good	Good	Good	Good

Sensitivity analysis meta-analysis

Figure S2. Forest plots of sensitivity analysis including studies excluded in the primary quantitative synthesis (not rated as good quality).

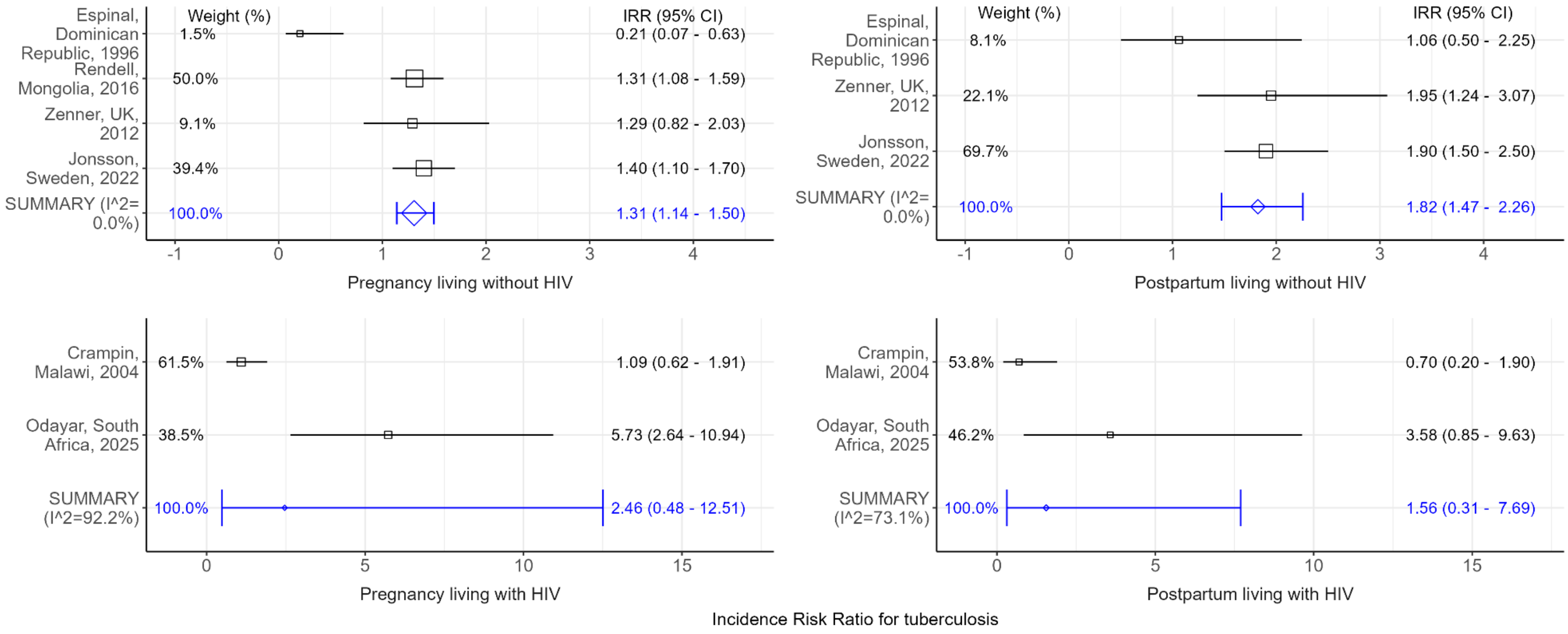

Population and births

Table S2. Population of women 15-49 years and estimated number of births in 2023.

	Mean (95% uncertainty interval)	
**WHO region**	**Population**	**Births**
Africa	397,213,400 (396,908,300, 397,518,500)	46,345,600 (45,923,700, 46,767,500)
Americas	291,867,300 (291,785,100, 291,949,500)	13,263,300 (12,777,000, 13,749,700)
Europe	186,746,900 (186,520,200, 186,973,500)	6,247,200 (6,034,700, 6,459,600)
SouthEast Asia	1,327,200,600 (1,326,791,000, 1,327,610,100)	65,316,400 (62,814,100, 67,818,700)
Western Pacific Region	12,340,600 (12,322,800, 12,358,300)	694,400 (659,800, 729,000)
Total	2,215,368,800 (2,214,803,700, 2,215,933,800)	131,866,900 (129,274,100, 134,459,600)

Person-time

Table S3. Estimated person-time during pregnancy and the postpartum period in 2023.

	Mean (95% uncertainty interval)	
**WHO region**	**Pregnancy**	**Postpartum**
Africa	35,552,800 (34,385,500, 36,720,000)	11,586,400 (11,206,000, 11,966,800)
Americas	10,174,600 (9,620,900, 10,728,300)	3,315,800 (3,135,400, 3,496,300)
Europe	4,792,300 (4,511,900, 5,072,800)	1,561,800 (1,470,400, 1,653,200)
SouthEast Asia	50,105,700 (46,333,400, 53,878,000)	16,329,100 (15,099,700, 17,558,500)
Western Pacific Region	532,700 (481,200, 584,200)	173,600 (156,800, 190,400)
Total	101,158,100 (97,160,600, 105,155,700)	32,966,700 (31,663,900, 34,269,500)

Estimates in high TB burden settings

Table S4. Estimates of tuberculosis incidence during pregnancy and postpartum disaggregated by HIV status for high TB burden countries. HIV; human immunodeficiency virus.

		Mean (95% uncertainty interval)	
**WHO**	**Pregnancy**		**Postpartum**

region	All pregnant women	Women living without HIV	Women living with HIV	All woman in the postpartum period	Women living without HIV	Women living with HIV
Angola	6,000 (3,201, 8,799)	3,201 (8,799, 4,487)	8,799 (4,487, 1,874)	4,487 (1,874, 7,100)	1,874 (7,100, 1,513)	7,100 (1,513, 1,306)
Bangladesh	5,578 (2,854, 8,303)	2,854 (8,303, 5,543)	8,303 (5,543, 2,544)	5,543 (2,544, 8,541)	2,544 (8,541, 36)	8,541 (36, 31)
Brazil	2,014 (1,782, 2,246)	1,782 (2,246, 635)	2,246 (635, 635)	635 (635, 635)	635 (635, 1,379)	635 (1,379, 1,379)
Cambodia	495 (203, 788)	203 (788, 488)	788 (488, 194)	488 (194, 782)	194 (782, 8)	782 (8, 7)
Central African Republic	2,377 (1,686, 3,068)	1,686 (3,068, 981)	3,068 (981, 981)	981 (981, 981)	981 (981, 1,396)	981 (1,396, 1,396)
China	3,697 (2,869, 4,525)	2,869 (4,525, 2,830)	4,525 (2,830, 2,830)	2,830 (2,830, 2,830)	2,830 (2,830, 867)	2,830 (867, 867)
Ethiopia	7,682 (4,051, 11,312)	4,051 (11,312, 6,887)	11,312 (6,887, 3,303)	6,887 (3,303, 10,472)	3,303 (10,472, 794)	10,472 (794, 697)
India	51,163 (36,012, 66,314)	36,012 (66,314, 49,899)	66,314 (49,899, 34,267)	49,899 (34,267, 65,532)	34,267 (65,532, 1,264)	65,532 (1,264, 1,171)
Indonesia	15,374 (12,628, 18,121)	12,628 (18,121, 13,827)	18,121 (13,827, 8,670)	13,827 (8,670, 18,983)	8,670 (18,983, 1,548)	18,983 (1,548, 1,412)
Kenya	4,258 (2,650, 5,866)	2,650 (5,866, 2,140)	5,866 (2,140, 720)	2,140 (720, 3,561)	720 (3,561, 2,118)	3,561 (2,118, 1,788)
Lesotho	414 (303, 525)	303 (525, 117)	525 (117, 39)	117 (39, 195)	39 (195, 297)	195 (297, 250)
Liberia	592 (361, 823)	361 (823, 398)	823 (398, 187)	398 (187, 608)	187 (608, 194)	608 (194, 170)
Mozambique	7,178 (4,402, 9,954)	4,402 (9,954, 3,866)	9,954 (3,866, 1,549)	3,866 (1,549, 6,184)	1,549 (6,184, 3,311)	6,184 (3,311, 2,846)
Namibia	374 (205, 544)	205 (544, 263)	544 (263, 263)	263 (263, 263)	263 (263, 112)	263 (112, 112)
Nigeria	18,402 (9,586, 27,218)	9,586 (27,218, 16,044)	27,218 (16,044, 7,489)	16,044 (7,489, 24,598)	7,489 (24,598, 2,358)	24,598 (2,358, 2,063)
Pakistan	16,292 (7,991, 24,594)	7,991 (24,594, 16,113)	24,594 (16,113, 475)	16,113 (475, 31,750)	475 (31,750, 180)	31,750 (180, 139)
Papua New Guinea	1,442 (1,007, 1,878)	1,007 (1,878, 1,209)	1,878 (1,209, 786)	1,209 (786, 1,631)	786 (1,631, 234)	1,631 (234, 215)
Philippines	6,535 (1,897, 11,172)	1,897 (11,172, 6,236)	11,172 (6,236, 1,626)	6,236 (1,626, 10,845)	1,626 (10,845, 299)	10,845 (299, 247)
Sierra Leone	1,107 (716, 1,498)	716 (1,498, 577)	1,498 (577, 251)	577 (251, 903)	251 (903, 530)	903 (530, 460)
South	13,398 (10,767,	10,767 (16,028,	16,028 (2,034,	2,034 (892,	892 (3,175,	3,175 (11,364,

Africa	16,028)	2,034)	892)	3,175)	11,364)	9,868)
Thailand	573 (336, 811)	336 (811, 452)	811 (452, 204)	452 (204, 701)	204 (701, 121)	701 (121, 106)
Zambia	2,281 (1,490, 3,072)	1,490 (3,072, 1,171)	3,072 (1,171, 522)	1,171 (522, 1,819)	522 (1,819, 1,110)	1,819 (1,110, 966)
Zimbabwe	2,424 (1,977, 2,871)	1,977 (2,871, 422)	2,871 (422, 190)	422 (190, 654)	190 (654, 2,002)	654 (2,002, 1,744)
Total	169,651 (148,356, 190,947)	148,356 (190,947, 136,617)	190,947 (136,617, 111,156)	136,617 (111,156, 162,077)	111,156 (162,077, 33,035)	162,077 (33,035, 31,353)

Global distribution of tuberculosis burden during pregnancy and postpartum

Figure S3. World map showing the estimated tuberculosis incidence during pregnancy by country.

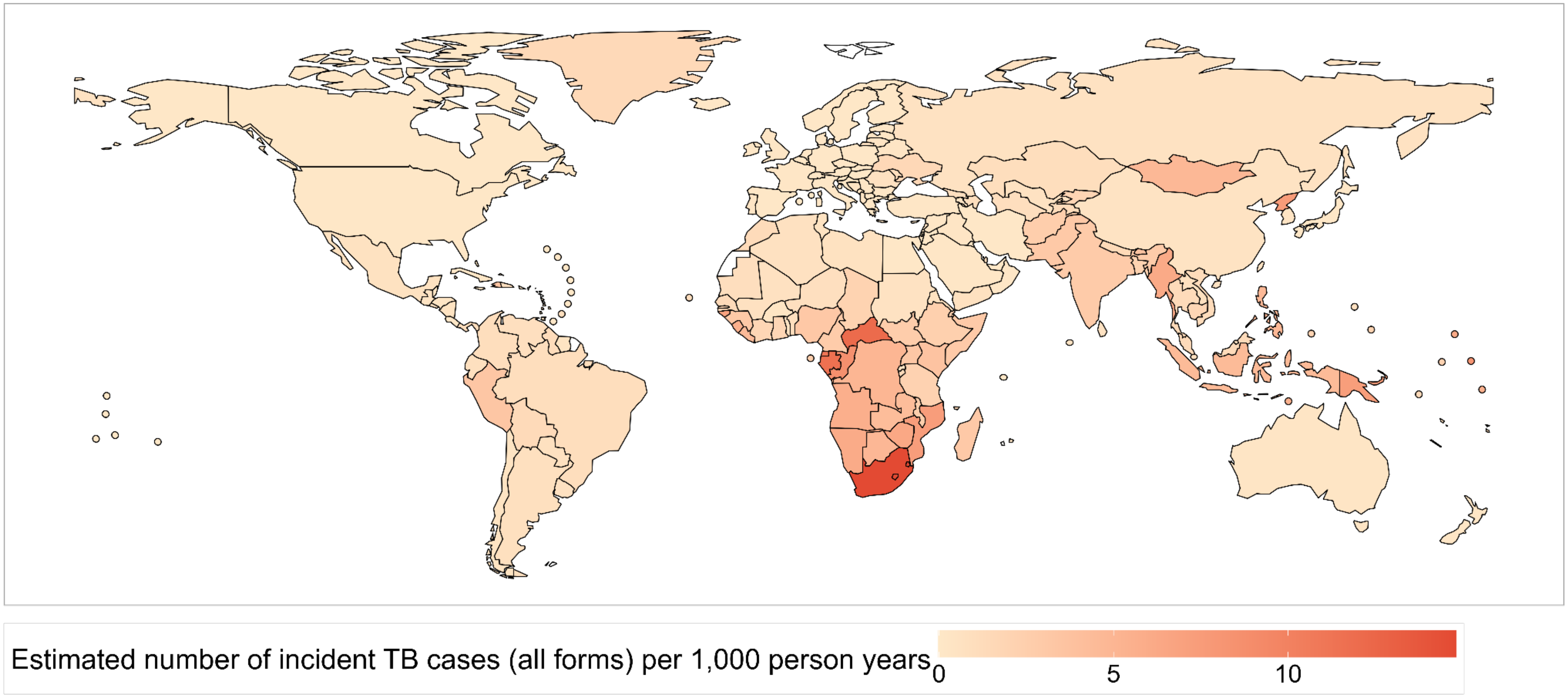

Figure S4. World map showing the estimated tuberculosis incidence during the postpartum period by country.

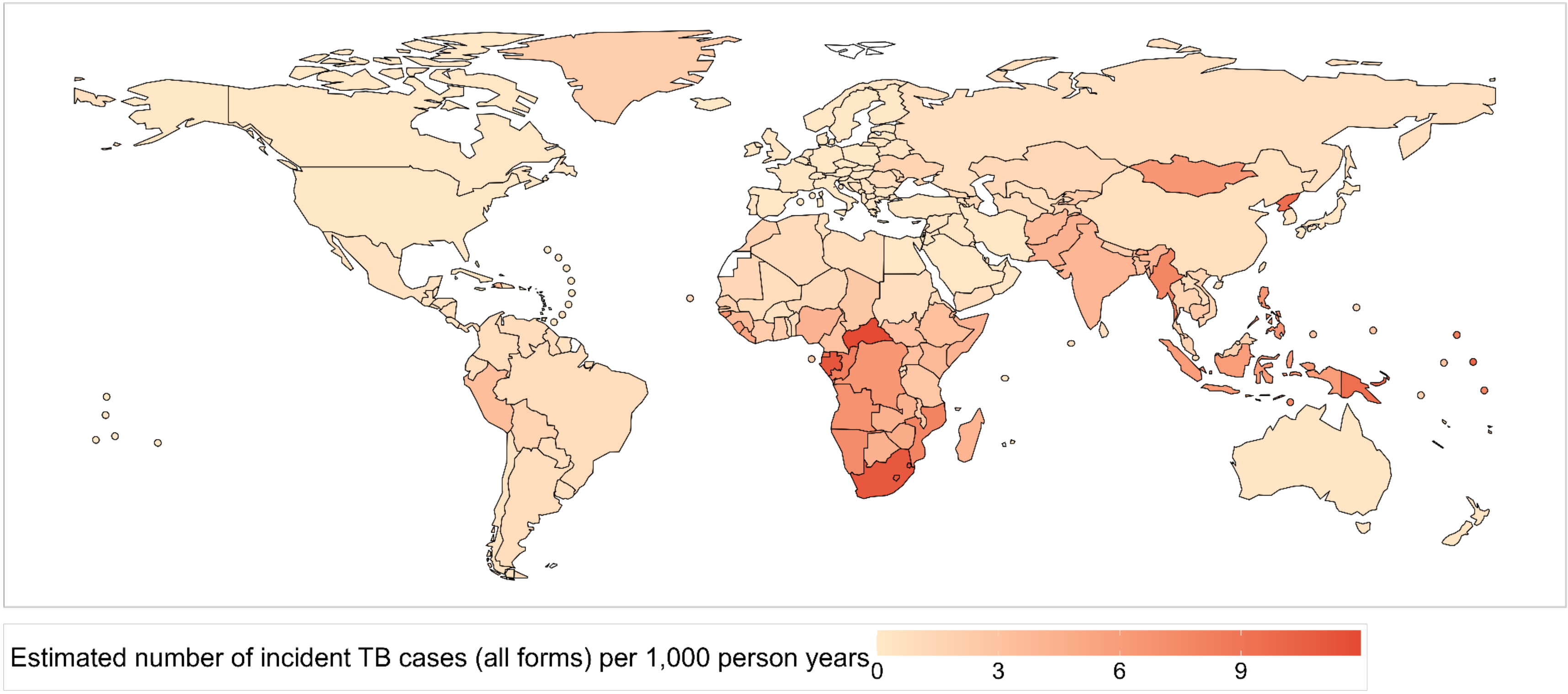

Country estimates

Table S5. Country level estimates of tuberculosis incidence during pregnancy and postpartum disaggregated by HIV status. HIV; human immunodeficiency virus

	Mean (95% uncertainty interval)					
	Pregnancy			Postpartum		
WHO region	Total	Women living without HIV	Women living with HIV	Total	Women living without HIV	Women living with HIV
Afghanistan	2,954 (1,232, 4,675)	2,950 (-73, 5,972)	4 (3, 5)	1,369 (808, 1,930)	1,368 (383, 2,353)	1 (0, 1)
Albania	5 (4, 6)	4 (4, 4)	1 (1, 1)	2 (2, 2)	2 (2, 2)	0 (0, 0)
Algeria	458 (235, 680)	437 (191, 684)	20 (17, 23)	207 (135, 279)	203 (123, 283)	4 (3, 5)
American Samoa	0 (0, 0)	0 (0, 0)	0 (0, 0)	0 (0, 0)	0 (0, 0)	0 (0, 0)
Andorra	0 (0, 0)	0 (0, 0)	0 (0, 0)	0 (0, 0)	0 (0, 0)	0 (0, 0)
Angola	6,000 (3,201, 8,799)	4,487 (1,874, 7,100)	1,513 (1,306, 1,720)	2,389 (1,477, 3,301)	2,081 (1,229, 2,933)	308 (241, 375)
Anguilla	0 (0, 0)	0 (0, 0)	0 (0, 0)	0 (0, 0)	0 (0, 0)	0 (0, 0)
Antigua & Barbuda	0 (0, 0)	0 (0, 0)	0 (0, 0)	0 (0, 0)	0 (0, 0)	0 (0, 0)
Argentina	310 (257, 364)	169 (169, 169)	141 (141, 141)	107 (90, 125)	79 (79, 79)	29 (29, 29)
Armenia	8 (6, 9)	4 (2, 6)	4 (3, 4)	3 (2, 3)	2 (1, 2)	1 (1, 1)
Aruba	0 (0, 0)	0 (0, 0)	0 (0, 0)	0 (0, 0)	0 (0, 0)	0 (0, 0)
Australia	46 (37, 55)	26 (26, 26)	20 (20, 20)	16 (13, 19)	12 (12, 12)	4 (4, 4)
Austria	4 (3, 5)	3 (3, 3)	1 (1, 1)	2 (1, 2)	1 (1, 1)	0 (0, 0)
Azerbaijan	85 (38, 133)	79 (30, 127)	6 (5, 7)	38 (22, 53)	37 (21, 52)	1 (1, 2)
Bahamas	0 (0, 1)	0 (0, 0)	0 (0, 0)	0 (0, 0)	0 (0, 0)	0 (0, 0)
Bahrain	7 (6, 8)	3 (3, 3)	4 (4, 4)	2 (2, 3)	1 (1, 1)	1 (1, 1)
Bangladesh	5,578 (2,854, 8,303)	5,543 (2,544, 8,541)	36 (31, 40)	2,578 (1,690, 3,466)	2,571 (1,593, 3,548)	7 (6, 9)
Barbados	0 (0, 0)	0 (0, 0)	0 (0, 0)	0 (0, 0)	0 (0, 0)	0 (0, 0)
Belarus	12 (8, 16)	8 (8, 8)	4 (4, 4)	5 (3, 6)	4 (4, 4)	1 (1, 1)
Belgium	10 (8, 12)	8 (8, 8)	2 (2, 2)	4 (3, 5)	4 (4, 4)	0 (0, 0)
Belize	1 (1, 2)	1 (0, 1)	1 (1, 1)	0 (0, 1)	0 (0, 0)	0 (0, 0)

Benin	330 (202, 457)	201 (88, 313)	129 (112, 146)	119 (78, 161)	93 (56, 130)	26 (21, 32)
Bermuda	0 (0, 0)	0 (0, 0)	0 (0, 0)	0 (0, 0)	0 (0, 0)	0 (0, 0)
Bhutan	28 (18, 38)	27 (27, 27)	1 (1, 1)	13 (9, 16)	12 (12, 12)	0 (0, 0)
Bolivia	271 (145, 397)	207 (89, 326)	64 (55, 72)	109 (68, 150)	96 (57, 135)	13 (10, 16)
Bosnia & Herzegovina	8 (5, 11)	6 (6, 6)	2 (2, 2)	3 (2, 4)	3 (3, 3)	0 (0, 0)
Botswana	227 (167, 287)	82 (35, 128)	145 (126, 165)	67 (48, 87)	38 (23, 53)	30 (23, 36)
Brazil	2,014 (1,782, 2,246)	635 (635, 635)	1,379 (1,379, 1,379)	575 (500, 651)	295 (295, 295)	281 (281, 281)
British Virgin Islands	0 (0, 0)	0 (0, 0)	0 (0, 0)	0 (0, 0)	0 (0, 0)	0 (0, 0)
Brunei	4 (3, 4)	2 (2, 2)	2 (2, 2)	1 (1, 2)	1 (1, 1)	0 (0, 0)
Bulgaria	10 (6, 13)	8 (8, 8)	2 (2, 2)	4 (3, 5)	4 (4, 4)	0 (0, 0)
Burkina Faso	196 (111, 281)	156 (75, 236)	40 (35, 45)	80 (53, 108)	72 (46, 99)	8 (7, 10)
Burundi	469 (226, 711)	416 (179, 654)	52 (45, 59)	204 (125, 283)	193 (116, 271)	11 (8, 13)
Cambodia	495 (203, 788)	488 (194, 782)	8 (7, 9)	228 (133, 323)	226 (130, 322)	2 (1, 2)
Cameroon	2,158 (1,353, 2,963)	1,137 (1,137, 1,137)	1,021 (1,021, 1,021)	735 (473, 997)	527 (527, 527)	208 (208, 208)
Canada	37 (30, 44)	18 (18, 18)	19 (19, 19)	12 (10, 15)	9 (9, 9)	4 (4, 4)
Cape Verde	5 (4, 6)	3 (1, 4)	2 (2, 2)	2 (1, 2)	1 (1, 2)	0 (0, 1)
Cayman Islands	0 (0, 0)	0 (0, 0)	0 (0, 0)	0 (0, 0)	0 (0, 0)	0 (0, 0)
Central African Republic	2,377 (1,686, 3,068)	981 (981, 981)	1,396 (1,396, 1,396)	739 (514, 964)	455 (455, 455)	284 (284, 284)
Chad	1,429 (827, 2,031)	1,062 (498, 1,626)	367 (321, 412)	567 (371, 764)	493 (309, 677)	75 (60, 90)
Chile	55 (48, 63)	18 (18, 18)	38 (38, 38)	16 (13, 18)	8 (8, 8)	8 (8, 8)
China	3,697 (2,869, 4,525)	2,830 (2,830, 2,830)	867 (867, 867)	1,489 (1,219, 1,759)	1,313 (1,313, 1,313)	176 (176, 176)
Colombia	550 (440, 660)	132 (132, 132)	418 (418, 418)	146 (110, 182)	61 (61, 61)	85 (85, 85)
Comoros	30 (25, 35)	2 (2, 2)	27 (27, 27)	7 (5, 8)	1 (1, 1)	6 (6, 6)
Congo - Brazzaville	1,272 (883, 1,660)	521 (207, 835)	751 (645, 857)	394 (268, 521)	241 (139, 344)	153 (118, 187)
Congo - Kinshasa	17,015 (9,121, 24,908)	14,303 (6,633, 21,974)	2,711 (2,370, 3,052)	7,186 (4,613, 9,758)	6,634 (4,134, 9,134)	552 (441, 663)
Cook Islands	0 (0, 0)	0 (0, 0)	0 (0, 0)	0 (0, 0)	0 (0, 0)	0 (0, 0)
Costa Rica	5 (4, 7)	3 (2, 4)	2 (2, 3)	2 (1, 2)	1 (1, 2)	0 (0, 1)

Croatia	1 (1, 1)	1 (1, 1)	1 (1, 1)	0 (0, 0)	0 (0, 0)	0 (0, 0)
Cuba	5 (4, 6)	3 (3, 4)	2 (1, 2)	2 (2, 2)	2 (1, 2)	0 (0, 0)
Curaçao	0 (0, 0)	0 (0, 0)	0 (0, 0)	0 (0, 0)	0 (0, 0)	0 (0, 0)
Cyprus	2 (2, 2)	1 (1, 1)	1 (1, 1)	1 (1, 1)	0 (0, 0)	0 (0, 0)
Czechia	9 (8, 10)	1 (1, 1)	8 (8, 8)	2 (2, 3)	1 (1, 1)	2 (2, 2)
Côte d'Ivoire	1,378 (716, 2,040)	1,085 (460, 1,711)	292 (253, 332)	563 (347, 779)	503 (299, 707)	59 (47, 72)
Denmark	3 (3, 4)	2 (2, 2)	1 (1, 1)	1 (1, 1)	1 (1, 1)	0 (0, 0)
Djibouti	45 (26, 63)	42 (22, 62)	3 (3, 3)	20 (14, 26)	19 (13, 26)	1 (0, 1)
Dominica	0 (0, 0)	0 (0, 0)	0 (0, 0)	0 (0, 0)	0 (0, 0)	0 (0, 0)
Dominican Republic	115 (84, 146)	55 (55, 55)	60 (60, 60)	38 (28, 48)	26 (26, 26)	12 (12, 12)
Ecuador	168 (125, 211)	93 (56, 130)	75 (68, 82)	58 (44, 72)	43 (31, 55)	15 (13, 17)
Egypt	215 (166, 265)	209 (154, 264)	6 (6, 7)	98 (82, 114)	97 (79, 115)	1 (1, 1)
El Salvador	35 (25, 44)	29 (19, 39)	5 (5, 6)	15 (11, 18)	14 (10, 17)	1 (1, 1)
Equatorial Guinea	494 (384, 605)	116 (116, 116)	378 (378, 378)	131 (95, 167)	54 (54, 54)	77 (77, 77)
Eritrea	48 (5, 91)	46 (3, 89)	1 (1, 2)	22 (8, 36)	21 (7, 35)	0 (0, 0)
Estonia	1 (1, 1)	1 (1, 1)	0 (0, 0)	0 (0, 0)	0 (0, 0)	0 (0, 0)
Eswatini	224 (164, 283)	47 (12, 82)	176 (146, 207)	58 (39, 77)	22 (10, 33)	36 (26, 46)
Ethiopia	7,682 (4,051, 11,312)	6,887 (3,303, 10,472)	794 (697, 891)	3,356 (2,173, 4,539)	3,194 (2,026, 4,363)	162 (130, 193)
Fiji	15 (11, 18)	5 (3, 8)	9 (8, 10)	4 (3, 5)	3 (2, 3)	2 (1, 2)
Finland	3 (2, 4)	2 (2, 2)	1 (1, 1)	1 (1, 1)	1 (1, 1)	0 (0, 0)
France	76 (61, 91)	61 (61, 61)	15 (15, 15)	31 (26, 36)	28 (28, 28)	3 (3, 3)
French Polynesia	1 (0, 1)	1 (1, 1)	0 (0, 0)	0 (0, 0)	0 (0, 0)	0 (0, 0)
Gabon	608 (422, 794)	260 (101, 419)	348 (298, 398)	191 (131, 252)	120 (69, 172)	71 (55, 87)
Gambia	120 (76, 164)	85 (44, 126)	35 (31, 39)	47 (32, 61)	40 (26, 53)	7 (6, 8)
Georgia	28 (19, 38)	25 (14, 36)	4 (3, 4)	12 (9, 16)	12 (8, 15)	1 (1, 1)
Germany	59 (50, 68)	37 (37, 37)	22 (22, 22)	22 (19, 25)	17 (17, 17)	4 (4, 4)
Ghana	1,234 (483, 1,984)	893 (202, 1,585)	341 (279, 403)	484 (239, 728)	414 (189, 640)	69 (49, 90)
Greece	4 (3, 4)	2 (2, 2)	1 (1, 1)	1 (1, 2)	1 (1, 1)	0 (0, 0)
Greenland	1 (1, 1)	1 (1, 1)	0 (0, 0)	0 (0, 0)	0 (0, 0)	0 (0, 0)

Grenada	0 (0, 0)	0 (0, 0)	0 (0, 0)	0 (0, 0)	0 (0, 0)	0 (0, 0)
Guam	1 (1, 2)	1 (1, 1)	0 (0, 0)	1 (1, 1)	1 (1, 1)	0 (0, 0)
Guatemala	106 (70, 142)	76 (43, 109)	30 (27, 33)	41 (30, 53)	35 (24, 46)	6 (5, 7)
Guinea	1,155 (688, 1,622)	736 (320, 1,151)	419 (364, 475)	427 (274, 579)	341 (206, 477)	85 (67, 103)
Guinea-Bissau	341 (236, 447)	160 (76, 245)	181 (159, 204)	111 (77, 146)	74 (47, 102)	37 (30, 44)
Guyana	15 (12, 17)	5 (3, 7)	10 (9, 11)	4 (3, 5)	2 (2, 3)	2 (2, 2)
Haiti	685 (486, 884)	377 (208, 547)	308 (275, 340)	238 (173, 303)	175 (120, 230)	63 (52, 73)
Honduras	56 (37, 75)	43 (25, 62)	13 (11, 14)	23 (16, 29)	20 (14, 26)	3 (2, 3)
Hong Kong SAR China	13 (10, 17)	13 (13, 13)	1 (1, 1)	6 (5, 7)	6 (6, 6)	0 (0, 0)
Hungary	4 (3, 5)	2 (2, 2)	2 (2, 2)	1 (1, 2)	1 (1, 1)	0 (0, 0)
Iceland	0 (0, 0)	0 (0, 0)	0 (0, 0)	0 (0, 0)	0 (0, 0)	0 (0, 0)
India	51,163 (36,012, 66,314)	49,899 (34,267, 65,532)	1,264 (1,171, 1,356)	23,401 (18,463, 28,338)	23,144 (18,049, 28,238)	257 (227, 287)
Indonesia	15,374 (12,628, 18,121)	13,827 (8,670, 18,983)	1,548 (1,412, 1,683)	6,728 (5,833, 7,623)	6,413 (4,732, 8,093)	315 (271, 359)
Iran	83 (48, 118)	77 (37, 117)	6 (5, 7)	37 (26, 48)	36 (23, 49)	1 (1, 1)
Iraq	225 (156, 294)	224 (224, 224)	1 (1, 1)	104 (82, 127)	104 (104, 104)	0 (0, 0)
Ireland	4 (3, 5)	3 (3, 3)	1 (1, 1)	2 (1, 2)	1 (1, 1)	0 (0, 0)
Israel	8 (7, 10)	5 (5, 5)	3 (3, 3)	3 (2, 4)	2 (2, 2)	1 (1, 1)
Italy	25 (20, 30)	18 (18, 18)	7 (7, 7)	10 (8, 11)	8 (8, 8)	2 (2, 2)
Jamaica	1 (1, 1)	0 (0, 1)	1 (0, 1)	0 (0, 0)	0 (0, 0)	0 (0, 0)
Japan	100 (90, 110)	32 (32, 32)	68 (68, 68)	29 (25, 32)	15 (15, 15)	14 (14, 14)
Jordan	13 (6, 20)	13 (13, 13)	0 (0, 0)	6 (4, 8)	6 (6, 6)	0 (0, 0)
Kazakhstan	347 (160, 535)	246 (246, 246)	102 (102, 102)	135 (74, 196)	114 (114, 114)	21 (21, 21)
Kenya	4,258 (2,650, 5,866)	2,140 (720, 3,561)	2,118 (1,788, 2,447)	1,424 (900, 1,948)	993 (530, 1,456)	431 (324, 538)
Kiribati	19 (11, 27)	17 (17, 17)	2 (2, 2)	8 (6, 11)	8 (8, 8)	0 (0, 0)
Kuwait	8 (6, 10)	7 (7, 7)	1 (1, 1)	4 (3, 4)	3 (3, 3)	0 (0, 0)
Kyrgyzstan	219 (145, 293)	174 (103, 246)	45 (40, 49)	90 (66, 114)	81 (58, 104)	9 (8, 10)
Laos	89 (46, 133)	71 (30, 113)	18 (15, 20)	37 (23, 51)	33 (20, 47)	4 (3, 4)

Latvia	2 (2, 3)	2 (2, 2)	1 (1, 1)	1 (1, 1)	1 (1, 1)	0 (0, 0)
Lebanon	25 (20, 31)	20 (20, 20)	5 (5, 5)	11 (9, 12)	10 (10, 10)	1 (1, 1)
Lesotho	414 (303, 525)	117 (39, 195)	297 (250, 343)	115 (78, 151)	54 (29, 80)	60 (45, 76)
Liberia	592 (361, 823)	398 (187, 608)	194 (170, 218)	224 (149, 299)	185 (116, 253)	40 (32, 47)
Libya	59 (28, 91)	53 (21, 84)	7 (6, 8)	26 (15, 36)	24 (14, 35)	1 (1, 2)
Lithuania	5 (4, 6)	3 (3, 3)	2 (2, 2)	2 (2, 2)	1 (1, 1)	0 (0, 0)
Luxembourg	1 (1, 1)	1 (1, 1)	0 (0, 0)	0 (0, 0)	0 (0, 0)	0 (0, 0)
Macao SAR China	1 (1, 2)	1 (1, 1)	0 (0, 0)	1 (0, 1)	1 (1, 1)	0 (0, 0)
Madagascar	2,351 (1,129, 3,573)	2,174 (931, 3,416)	177 (153, 201)	1,044 (646, 1,443)	1,008 (603, 1,413)	36 (28, 44)
Malawi	1,294 (839, 1,748)	429 (120, 738)	865 (719, 1,011)	375 (227, 523)	199 (98, 300)	176 (128, 224)
Malaysia	447 (241, 652)	383 (184, 583)	64 (56, 71)	191 (124, 258)	178 (113, 243)	13 (10, 15)
Maldives	2 (1, 2)	2 (2, 2)	0 (0, 0)	1 (1, 1)	1 (1, 1)	0 (0, 0)
Mali	498 (256, 740)	430 (193, 667)	69 (60, 78)	213 (134, 292)	199 (122, 277)	14 (11, 17)
Malta	1 (1, 1)	0 (0, 0)	0 (0, 0)	0 (0, 0)	0 (0, 0)	0 (0, 0)
Marshall Islands	3 (2, 5)	3 (3, 3)	0 (0, 0)	2 (1, 2)	2 (2, 2)	0 (0, 0)
Mauritania	128 (59, 197)	121 (50, 191)	7 (6, 8)	58 (35, 80)	56 (33, 79)	1 (1, 2)
Mauritius	2 (2, 3)	1 (0, 1)	1 (1, 2)	1 (1, 1)	0 (0, 0)	0 (0, 0)
Mexico	978 (749, 1,207)	237 (237, 237)	741 (741, 741)	261 (186, 335)	110 (110, 110)	151 (151, 151)
Micronesia (Federated States of)	4 (2, 5)	4 (4, 4)	0 (0, 0)	2 (1, 2)	2 (2, 2)	0 (0, 0)
Moldova	22 (19, 26)	12 (9, 16)	10 (10, 11)	8 (7, 9)	6 (5, 7)	2 (2, 2)
Monaco	0 (0, 0)	0 (0, 0)	0 (0, 0)	0 (0, 0)	0 (0, 0)	0 (0, 0)
Mongolia	243 (110, 376)	206 (206, 206)	37 (37, 37)	103 (60, 146)	96 (96, 96)	8 (8, 8)
Montenegro	1 (1, 1)	0 (0, 0)	1 (1, 1)	0 (0, 0)	0 (0, 0)	0 (0, 0)
Montserrat	0 (0, 0)	0 (0, 0)	0 (0, 0)	0 (0, 0)	0 (0, 0)	0 (0, 0)
Morocco	629 (464, 794)	594 (322, 867)	35 (31, 39)	283 (229, 337)	276 (187, 364)	7 (6, 8)
Mozambique	7,178 (4,402, 9,954)	3,866 (1,549, 6,184)	3,311 (2,846, 3,777)	2,467 (1,562, 3,372)	1,793 (1,038, 2,548)	674 (522, 826)
Myanmar (Burma)	4,215 (2,011, 6,419)	3,332 (1,231, 5,433)	883 (753, 1,014)	1,725 (1,007, 2,443)	1,545 (861, 2,230)	180 (137, 222)

Namibia	374 (205, 544)	263 (263, 263)	112 (112, 112)	145 (90, 200)	122 (122, 122)	23 (23, 23)
Nauru	0 (0, 1)	0 (0, 0)	0 (0, 0)	0 (0, 0)	0 (0, 0)	0 (0, 0)
Nepal	620 (212, 1,027)	606 (198, 1,015)	13 (11, 15)	284 (151, 417)	281 (148, 414)	3 (2, 3)
Netherlands	15 (12, 18)	9 (9, 9)	7 (7, 7)	5 (4, 6)	4 (4, 4)	1 (1, 1)
New Caledonia	1 (1, 1)	1 (1, 1)	0 (0, 0)	0 (0, 0)	0 (0, 0)	0 (0, 0)
New Zealand	6 (5, 8)	5 (5, 5)	1 (1, 1)	3 (2, 3)	2 (2, 2)	0 (0, 0)
Nicaragua	54 (34, 74)	40 (21, 60)	13 (12, 15)	21 (15, 28)	19 (12, 25)	3 (2, 3)
Niger	749 (363, 1,135)	686 (305, 1,067)	63 (55, 71)	331 (205, 457)	318 (194, 442)	13 (10, 15)
Nigeria	18,402 (9,586, 27,218)	16,044 (7,489, 24,598)	2,358 (2,063, 2,653)	7,921 (5,048, 10,794)	7,441 (4,653, 10,229)	480 (384, 576)
Niue	0 (0, 0)	0 (0, 0)	0 (0, 0)	0 (0, 0)	0 (0, 0)	0 (0, 0)
North Korea	2,061 (1,522, 2,601)	1,576 (1,576, 1,576)	485 (485, 485)	830 (654, 1,006)	731 (731, 731)	99 (99, 99)
North Macedonia	2 (1, 3)	1 (1, 1)	1 (1, 1)	1 (0, 1)	0 (0, 0)	0 (0, 0)
Northern Mariana Islands	0 (0, 0)	0 (0, 0)	0 (0, 0)	0 (0, 0)	0 (0, 0)	0 (0, 0)
Norway	4 (3, 5)	3 (3, 3)	1 (1, 1)	2 (1, 2)	1 (1, 1)	0 (0, 0)
Oman	10 (8, 12)	9 (9, 9)	1 (1, 1)	4 (4, 5)	4 (4, 4)	0 (0, 0)
Pakistan	16,292 (7,991, 24,594)	16,113 (475, 31,750)	180 (139, 221)	7,510 (4,804, 10,215)	7,473 (2,377, 12,569)	37 (23, 50)
Palau	0 (0, 0)	0 (0, 0)	0 (0, 0)	0 (0, 0)	0 (0, 0)	0 (0, 0)
Palestinian Territories	0 (0, 0)	0 (0, 0)	0 (0, 0)	0 (0, 0)	0 (0, 0)	0 (0, 0)
Panama	56 (43, 69)	24 (14, 35)	32 (29, 35)	18 (13, 22)	11 (8, 15)	6 (5, 8)
Papua New Guinea	1,442 (1,007, 1,878)	1,209 (786, 1,631)	234 (215, 253)	608 (466, 750)	561 (423, 698)	48 (41, 54)
Paraguay	75 (63, 87)	49 (37, 61)	26 (25, 28)	28 (24, 32)	23 (19, 27)	5 (5, 6)
Peru	1,405 (943, 1,867)	649 (649, 649)	756 (756, 756)	455 (304, 605)	301 (301, 301)	154 (154, 154)
Philippines	6,535 (1,897, 11,172)	6,236 (1,626, 10,845)	299 (247, 351)	2,953 (1,442, 4,464)	2,892 (1,390, 4,394)	61 (44, 78)
Poland	23 (18, 28)	16 (16, 16)	6 (6, 6)	9 (7, 10)	8 (8, 8)	1 (1, 1)
Portugal	16 (13, 19)	11 (11, 11)	5 (5, 5)	6 (5, 7)	5 (5, 5)	1 (1, 1)
Puerto Rico	0 (0, 0)	0 (0, 0)	0 (0, 0)	0 (0, 0)	0 (0, 0)	0 (0, 0)

Qatar	16 (13, 20)	15 (15, 15)	1 (1, 1)	7 (6, 8)	7 (7, 7)	0 (0, 0)
Romania	92 (75, 109)	67 (50, 84)	25 (24, 26)	36 (31, 42)	31 (26, 37)	5 (5, 6)
Russia	492 (265, 718)	339 (339, 339)	153 (153, 153)	188 (115, 262)	157 (157, 157)	31 (31, 31)
Rwanda	145 (100, 190)	115 (72, 157)	30 (28, 33)	59 (45, 74)	53 (39, 67)	6 (5, 7)
Samoa	0 (0, 0)	0 (0, 0)	0 (0, 0)	0 (0, 0)	0 (0, 0)	0 (0, 0)
San Marino	0 (0, 0)	0 (0, 0)	0 (0, 0)	0 (0, 0)	0 (0, 0)	0 (0, 0)
Saudi Arabia	67 (59, 75)	40 (40, 40)	27 (27, 27)	24 (22, 27)	19 (19, 19)	5 (5, 5)
Senegal	562 (319, 806)	507 (266, 747)	56 (49, 62)	246 (167, 326)	235 (157, 313)	11 (9, 13)
Serbia	8 (6, 11)	7 (7, 7)	1 (1, 1)	3 (3, 4)	3 (3, 3)	0 (0, 0)
Seychelles	0 (0, 0)	0 (0, 0)	0 (0, 0)	0 (0, 0)	0 (0, 0)	0 (0, 0)
Sierra Leone	1,107 (716, 1,498)	577 (251, 903)	530 (460, 600)	376 (248, 503)	268 (162, 374)	108 (85, 131)
Singapore	33 (26, 40)	19 (19, 19)	14 (14, 14)	12 (10, 14)	9 (9, 9)	3 (3, 3)
Sint Maarten	0 (0, 0)	0 (0, 0)	0 (0, 0)	0 (0, 0)	0 (0, 0)	0 (0, 0)
Slovakia	3 (2, 3)	1 (1, 1)	2 (2, 2)	1 (1, 1)	0 (0, 0)	0 (0, 0)
Slovenia	1 (1, 1)	0 (0, 0)	0 (0, 0)	0 (0, 0)	0 (0, 0)	0 (0, 0)
Solomon Islands	19 (12, 26)	17 (17, 17)	2 (2, 2)	8 (6, 11)	8 (8, 8)	0 (0, 0)
Somalia	1,842 (807, 2,878)	1,807 (759, 2,856)	35 (30, 40)	845 (508, 1,183)	838 (496, 1,180)	7 (6, 9)
South Africa	13,398 (10,767, 16,028)	2,034 (892, 3,175)	11,364 (9,868, 12,860)	3,256 (2,399, 4,113)	943 (571, 1,315)	2,313 (1,825, 2,800)
South Korea	94 (87, 101)	17 (17, 17)	77 (77, 77)	24 (21, 26)	8 (8, 8)	16 (16, 16)
South Sudan	699 (423, 975)	467 (217, 717)	232 (203, 261)	264 (174, 354)	217 (135, 298)	47 (38, 57)
Spain	30 (24, 35)	18 (18, 18)	12 (12, 12)	11 (9, 12)	8 (8, 8)	2 (2, 2)
Sri Lanka	143 (89, 198)	105 (105, 105)	39 (39, 39)	56 (39, 74)	49 (49, 49)	8 (8, 8)
St. Kitts & Nevis	0 (0, 0)	0 (0, 0)	0 (0, 0)	0 (0, 0)	0 (0, 0)	0 (0, 0)
St. Lucia	0 (0, 0)	0 (0, 0)	0 (0, 0)	0 (0, 0)	0 (0, 0)	0 (0, 0)
St. Vincent & Grenadines	1 (1, 1)	0 (0, 0)	0 (0, 0)	0 (0, 0)	0 (0, 0)	0 (0, 0)
Sudan	737 (316, 1,157)	673 (178, 1,167)	64 (53, 75)	325 (188, 462)	312 (151, 473)	13 (9, 17)
Suriname	4 (3, 5)	2 (1, 3)	2 (1, 2)	1 (1, 2)	1 (1, 1)	0 (0, 0)
Sweden	7 (5, 8)	5 (5, 5)	2 (2, 2)	3 (2, 3)	2 (2, 2)	0 (0, 0)

Switzerland	6 (5, 7)	4 (4, 4)	2 (2, 2)	2 (2, 3)	2 (2, 2)	0 (0, 0)
Syria	92 (56, 129)	79 (79, 79)	13 (13, 13)	39 (27, 51)	37 (37, 37)	3 (3, 3)
São Tomé & Príncipe	13 (9, 17)	3 (3, 3)	11 (11, 11)	3 (2, 5)	1 (1, 1)	2 (2, 2)
Tajikistan	241 (149, 333)	205 (114, 297)	35 (32, 39)	103 (73, 132)	95 (65, 125)	7 (6, 8)
Tanzania	3,985 (1,347, 6,624)	2,758 (367, 5,150)	1,227 (978, 1,477)	1,529 (669, 2,389)	1,279 (500, 2,059)	250 (168, 331)
Thailand	573 (336, 811)	452 (204, 701)	121 (106, 137)	234 (157, 312)	210 (129, 291)	25 (20, 30)
Timor-Leste	128 (54, 201)	120 (120, 120)	8 (8, 8)	57 (33, 81)	56 (56, 56)	2 (2, 2)
Togo	105 (76, 134)	75 (48, 103)	30 (27, 32)	41 (31, 50)	35 (26, 44)	6 (5, 7)
Tokelau	0 (0, 0)	0 (0, 0)	0 (0, 0)	0 (0, 0)	0 (0, 0)	0 (0, 0)
Tonga	0 (0, 0)	0 (0, 0)	0 (0, 0)	0 (0, 0)	0 (0, 0)	0 (0, 0)
Trinidad & Tobago	3 (2, 3)	2 (2, 2)	1 (1, 1)	1 (1, 1)	1 (1, 1)	0 (0, 0)
Tunisia	67 (37, 97)	62 (22, 102)	5 (4, 6)	30 (20, 40)	29 (16, 42)	1 (1, 1)
Turkey	173 (118, 228)	112 (112, 112)	61 (61, 61)	64 (46, 82)	52 (52, 52)	12 (12, 12)
Turkmenistan	129 (94, 165)	66 (66, 66)	63 (63, 63)	44 (32, 55)	31 (31, 31)	13 (13, 13)
Turks & Caicos Islands	0 (0, 0)	0 (0, 0)	0 (0, 0)	0 (0, 0)	0 (0, 0)	0 (0, 0)
Tuvalu	1 (1, 1)	1 (1, 1)	0 (0, 0)	0 (0, 0)	0 (0, 0)	0 (0, 0)
Uganda	4,370 (3,059, 5,682)	1,686 (728, 2,643)	2,684 (2,327, 3,042)	1,328 (901, 1,756)	782 (470, 1,094)	546 (430, 663)
Ukraine	218 (128, 307)	133 (133, 133)	85 (85, 85)	79 (50, 108)	62 (62, 62)	17 (17, 17)
United Arab Emirates	1 (1, 1)	0 (0, 0)	0 (0, 0)	0 (0, 0)	0 (0, 0)	0 (0, 0)
United Kingdom	91 (77, 106)	69 (69, 69)	23 (23, 23)	36 (32, 41)	32 (32, 32)	5 (5, 5)
United States	153 (127, 178)	78 (78, 78)	75 (75, 75)	51 (43, 60)	36 (36, 36)	15 (15, 15)
Uruguay	18 (15, 20)	9 (6, 11)	9 (8, 9)	6 (5, 7)	4 (3, 5)	2 (2, 2)
Uzbekistan	413 (175, 651)	384 (150, 619)	28 (24, 33)	184 (107, 262)	178 (102, 255)	6 (4, 7)
Vanuatu	4 (2, 5)	3 (3, 3)	0 (0, 0)	2 (1, 2)	2 (2, 2)	0 (0, 0)
Venezuela	283 (197, 370)	161 (70, 253)	122 (106, 139)	100 (72, 128)	75 (45, 105)	25 (20, 30)
Vietnam	1,236 (574, 1,898)	1,158 (504, 1,813)	78 (67, 88)	553 (337, 769)	537 (324, 750)	16 (12, 19)
Wallis & Futuna	0 (0, 0)	0 (0, 0)	0 (0, 0)	0 (0, 0)	0 (0, 0)	0 (0, 0)

Yemen	749 (578, 921)	736 (454, 1,017)	14 (12, 15)	344 (288, 400)	341 (249, 433)	3 (2, 3)
Zambia	2,281 (1,490, 3,072)	1,171 (522, 1,819)	1,110 (966, 1,254)	769 (511, 1,027)	543 (332, 754)	226 (179, 273)
Zimbabwe	2,424 (1,977, 2,871)	422 (190, 654)	2,002 (1,744, 2,261)	603 (458, 749)	196 (120, 271)	408 (323, 492)
Total	239,543 (216,311, 262,776)	187,838 (160,762, 214,915)	51,705 (49,915, 53,495)	97,643 (90,072, 105,215)	87,121 (78,297, 95,945)	10,523 (9,939, 11,106)

References

1 Abrams EJ, Jao J, Madlala HP, *et al.* An observational cohort study to investigate the impact of dolutegravir in pregnancy and its obesogenic effects on the metabolic health of women living with HIV and their children: Study protocol. *PloS One* 2024; **19**: e0307296.

2 Odayar J, Abrams EJ, Jao J, *et al.* Incidence of Tuberculosis in South African Women Living With HIV During Pregnancy and Postpartum. San Francisco, USA, 2025.

3 Global Tuberculosis Programme. Tuberculosis data. Data reported by countries to WHO and estimates of tuberculosis burden generated by WHO for the Global Tuberculosis Report. https://www.who.int/teams/global-tuberculosis-programme/data (accessed Oct 4, 2024).

4 UNAIDS DATA 2024. Geneva: Joint United Nations Programme on HIV/AIDS. 2024. https://www.unaids.org/en/regionscountries/countries/nigeria (accessed Jan 24, 2025).

5 US National Heart Lung and Blood Institute. Study Quality Assessment Tools. The NIH's Quality Assessment Tool for Observational Cohort and Cross-Sectional Studies. https://www.nhlbi.nih.gov/health-topics/study-quality-assessment-tools (accessed Oct 2, 2024).

6 Crampin AC, Glynn JR, Floyd S, *et al.* Tuberculosis and gender: exploring the patterns in a case control study in Malawi. *Int J Tuberc Lung Dis Off J Int Union Tuberc Lung Dis* 2004; **8**: 194–203.

7 Espinal MA, Reingold AL, Lavandera M. Effect of pregnancy on the risk of developing active tuberculosis. *J Infect Dis* 1996; **173**: 488–91.

8 Odayar J, Rangaka MX, Zerbe A, *et al.* Burden of tuberculosis in HIV-positive pregnant women in Cape Town, South Africa. *Int J Tuberc Lung Dis Off J Int Union Tuberc Lung Dis* 2018; **22**: 760–5.

9 Rendell NL, Batjargal N, Jadambaa N, Dobler CC. Risk of tuberculosis during pregnancy in Mongolia, a high incidence setting with low HIV prevalence. *Int J Tuberc Lung Dis Off J Int Union Tuberc Lung Dis* 2016; **20**: 1615–20.

10 Zenner D, Kruijshaar ME, Andrews N, Abubakar I. Risk of tuberculosis in pregnancy: a national, primary care-based cohort and self-controlled case series study. *Am J Respir Crit Care Med* 2012; **185**: 779–84.

11 Jonsson J, Kühlmann-Berenzon S, Berggren I, Bruchfeld J. Increased risk of active tuberculosis during pregnancy and postpartum: a register-based cohort study in Sweden. *Eur Respir J* 2020; **55**: 1901886.
